# A Case of Phenibut Withdrawal Management and Detoxification Using Baclofen in the Outpatient Setting

**DOI:** 10.1155/2024/8824770

**Published:** 2024-07-04

**Authors:** Emma DiFiore, Julie Pittman

**Affiliations:** Anne Burnett Marion School of Medicine Texas Christian University, Fort Worth, TX, USA

## Abstract

Phenibut, a GABA_B_ receptor agonist, has surged in popularity due to its nootropic and anxiolytic effects. Despite not being FDA approved, it is accessible online due to its marketing as a dietary supplement, leading to unregulated distribution. Increasing reports have highlighted the risks of addiction and severe withdrawal symptoms associated with phenibut use. This case report explores the management of phenibut withdrawal in an outpatient setting using a baclofen taper. The slow taper was complicated by various withdrawal symptoms, and the patient was ultimately stabilized on lorazepam, baclofen, gabapentin, and clonidine after 5-months time. This case is unique, as it also highlights challenges in tapering off baclofen following phenibut detoxification. The study underscores the need for further research on the pharmacological management of phenibut withdrawal, emphasizing the importance of raising awareness about phenibut's dangers and associated clinical presentations.

## 1. Introduction

Nootropic drugs such as phenibut have become popular in recent years due to their marketing as a “natural” alternative for cognitive enhancement. First discovered in Russia in the 1960s, phenibut primarily acts as an agonist on GABA_B_ receptors (and to a lesser extent GABA_A_ and *β*-phenethylamine receptors) to produce anxiolytic and nootropic effects [1]. While widely used in Russia for psychiatric disorders, phenibut is not FDA-approved in the United States. Despite this, phenibut is easily accessible through online purchasing due to its marketing as a dietary supplement. This allows for unregulated distribution of the drug in varying doses and formulations [2]. Over the years, a multitude of case reports have emerged exposing the dangers associated with phenibut use, including its propensity for addiction, potential for severe withdrawal, and hazardous cases of intoxication [3, 4].

Reported symptoms associated with phenibut withdrawal include hallucinations, anxiety, irritability, psychomotor agitation, hypertension, tachycardia, and even seizures [3, 4]. There are few case reports of phenibut taper and withdrawal management in the outpatient setting using baclofen [5, 6]. Additionally, there have been no reported cases of subsequent difficulty tapering off of baclofen itself postdetox of phenibut. To add to the growing literature, the following report highlights an outpatient case of phenibut detoxification using a baclofen taper and the challenges encountered.

## 2. Case Presentation

This case highlights a 54-year-old male with a past medical history of hypertension, chronic back pain, and depression who needed assistance with outpatient detoxification from phenibut. He first came across phenibut while searching online for a nootropic to help with “thinking and memory.“ He stated it initially worked well, however, he eventually developed a tolerance and increased his dosage up to 14 g/day. He attempted to self-taper but developed hallucinations and severe anxiety. He was hospitalized for 3 days due to withdrawal symptoms and was discharged on lorazepam. He reported no concurrent substance use at the time and was solely taking phenibut. Due to the COVID-19 pandemic occurring at this time, a referral for outpatient detoxification was made.

At the initial assessment, the patient was taking 8 g/day of phenibut and 3 mg/day of lorazepam. Over 5 months, he was able to slowly taper off phenibut completely in the outpatient setting (Table 1). He was initially started on a baclofen taper as well as both gabapentin and clonidine as adjuncts for withdrawal symptoms including anxiety, irritability, blood pressure spikes, and other hyperadrenergic symptoms. Once the patient reached the maximum dose of baclofen (80 mg/day), lorazepam was increased incrementally as phenibut was tapered to target withdrawal symptoms. Throughout this time, the taper was complicated by various symptoms including spikes in blood pressure, hallucinations, dizziness, akathisia, diaphoresis, irritability, and anxiety. The patient was stabilized on the following doses: lorazepam 6 mg QID (24 mg/day), baclofen 20 mg QID (80 mg/day), gabapentin 300 mg TID, and clonidine 0.1 mg TID PRN.

Once off of phenibut, lorazepam was slowly tapered over 14 months. He was taking up to 24 mg total daily of lorazepam at the height of his withdrawals during phenibut detoxification. The patient currently remains on baclofen 80 mg/day. Attempts to taper off of baclofen have been unsuccessful due to symptoms similar to what was experienced during withdrawal from phenibut (e.g., anxiety, irritability) but to a much lesser extent. For that reason, adjunctive therapies to target underlying anxiety, such as buspirone and fluoxetine, were initiated with some success. However, despite these difficulties with baclofen dependency, the patient has remained off of phenibut since 11/2020 and is content with this outcome.

## 3. Discussion

Different methods for phenibut detoxification have been highlighted in multiple case reports [5, 6, 7, 8, 9, 10] and literature reviews [3, 4]. In this particular case, baclofen was chosen for substitution during the phenibut detoxification due to its structural similarity to phenibut and its agonism on GABA_B_ receptors [1]. Gabapentin (decreased glutamate via *α*2*δ* subunit of voltage-dependent Ca^2+^ channels) and lorazepam (GABA_A_ agonist) were chosen as adjuncts for similar reasons [1, 3, 4, 11]. Clonidine was also used primarily for our patient's blood pressure spikes throughout detoxification. Of note, withdrawal symptoms of hypertension were similarly noted in a few case reports, but not nearly to the extent of what our patient experienced [7, 8]. It is possible our patient had underlying chronic hypertension or rebound hypertension from clonidine use.

As suggested by Samokhvalov et al. [5], the target dosage for phenibut detoxification should be 8–10 mg of baclofen substituted for every 1 g of phenibut. A few other case reports have shown success with baclofen at similar doses [7, 9]. Those who used baclofen at lower doses [8] or who did not substitute with baclofen at all [6] had less success tapering off of phenibut. One exception to these findings was seen in a case report by Coenen et al. [10], where their patient merely required 3 mg of baclofen per gram of phenibut for detoxification. It is important to note, however, that the patient was using other adjuncts such as diazepam and pregabalin. It is possible that the synergistic effect of these three medications allowed for a lower dose of baclofen.

Being that our patient initiated outpatient treatment while on 8 g/day of phenibut, this may explain why he had such difficulty with withdrawal symptoms early on in treatment while only on 15 mg of baclofen. It is also important to keep in mind that, prior to his admission to the hospital, he was taking up to 14 g/day of phenibut. To our knowledge, there has only been one other case report of a patient using phenibut at this large of a daily dose (up to 34.5 g/day) [10]. It is possible that this contributed to the withdrawal symptoms experienced throughout his taper despite being on polytherapy with baclofen, lorazepam, and gabapentin.

Some reports highlighted the use of adjuncts to baclofen during phenibut detoxification including citalopram, gabapentin, benzodiazepines, phenobarbital, acamprosate, and pregabalin [5, 8, 10]. Duration of detox also varied from 1 week up to 24 weeks regardless of the use of monotherapy with baclofen [7, 9], polytherapy with baclofen and other adjuncts [5, 8, 10], or no substitution therapy at all [6]. It is important to note that, unlike in our case, other substances besides phenibut were at play in certain case reports including alcohol, kratom, benzodiazepines, and methamphetamines to name a few. This makes it difficult to discern which combination of therapies were most successful.

Our patient was finally able to discontinue phenibut on baclofen 20 mg QID and lorazepam 6 mg QID. The risks of using these sedating medications in an outpatient setting were far outweighed by the benefits of assisting the patient by any means necessary for detoxification. The patient was heavily counseled on safety precautions including holding benzodiazepines for sedation and being unable to drive a vehicle until medically cleared. Despite such a high dose of lorazepam, the patient was able to taper off both the phenibut and benzodiazepine successfully.

It is unclear why the patient has struggled to taper off baclofen, as this was not an issue encountered in other case reports. One hypothesis considered is the modulation of GABA_B_ receptors—and to some extent GABA_A_ receptors [1]— from the patient's long-term, high-dose phenibut use leading to a vast rebound of GABAergic activity on cessation of medication. This mirrors the effect chronic benzodiazepine exposure has on GABA_A_ receptors, which too is postulated to cause withdrawal syndromes due to various mechanisms such as receptor downregulation, degradation of receptor subunits, and changes in receptor gene expression [12].

## 4. Conclusion

To our knowledge, this is the only case in current literature where a patient has had difficulty tapering off of baclofen following its use for phenibut detoxification. For this reason, it is prudent that further studies be published on the pharmacological management of phenibut withdrawal to determine which treatment methods are most favorable. We must also continue to raise awareness of the dangers associated with phenibut use and how withdrawal may present clinically.

## Figures and Tables

**Table 1:** Treatment summary.

Duration of taper (visit # and time elapsed)	Phenibut (g/day)	Baclofen (mg/day)	Lorazepam (mg/day)	Gabapentin (mg/day)	Clonidine (mg/day)	Propranolol (mg/day)	Symptoms
Visit 1	8 g	—	3 mg	—	—	—	Previously taking up to 14 g/day. Tried tapering on own and developed hallucinations, elevated BP, dizziness, and anxiety. Discharged from ED on lorazepam 1 mg TID. Pt reports feeling like he is “crawling out of skin”

Visit 2 (1 day)	8 g	15 mg (5 mg TID)	6 mg	900 mg (300 mg TID)	0.3 mg (0.1 mg TID)	—	Increased lorazepam from 1 mg TID to 2 mg TID due to “feeling uneasy.” BP still elevated but beginning to trend down

Visit 3 (4 days)	4 g	30 mg (10 mg TID)	6 mg	900 mg (300 mg TID)	0.3 mg (0.1 mg TID)	—	Decreased phenibut but “feeling sketchy” and feeling as though he is going to “blast off” similar to akathisia. BP is still elevated but stable (140 s systolic)

Visit 4 (6 days)	2 g	80 mg (20 mg QID)	24 mg	900 mg (300 mg TID)	—	30 mg (10 mg TID)	Increased lorazepam and overall feeling calmer. Began experiencing hypnopompic hallucinations of “beeps and dots.” Akathisia improved with propranolol. BP much improved without needing clonidine

Visit 5 (2 weeks)	4 g	80 mg (20 mg QID)	24 mg	900 mg (300 mg TID)	—	30 mg (10 mg TID)	Tried to decrease lorazepam to TID but could not tolerate. Ended up increasing phenibut back to 4 g due to withdrawal symptoms. Some episodes of elevated BP

Visit 6 (3.5 weeks)	3.5–4 g	80 mg (20 mg QID)	24 mg	—	0.3 mg (0.1 mg TID)	30 mg (10 mg TID)	Having difficult time decreasing phenibut. Feeling regret for trying it in the first place. Stopped taking gabapentin. BP stable (140 s systolic) on clonidine and propranolol

Visit 7 (6 weeks)	1 g	80 mg (20 mg QID)	24 mg	900 mg (300 mg TID)	0.3 mg (0.1 mg TID)	30 mg (10 mg TID)	Feeling irritable. Decreased phenibut from 3.5 g to 1 g over last 2 weeks (on 1 g for last 2 days). BP spiked to 190/120. Taking clonidine intermittently

Visit 8 (7.5 weeks)	0.5 g	80 mg (20 mg QID)	24 mg	900 mg (300 mg TID)	0.3 mg (0.1 mg TID)	30 mg (10 mg TID)	Experiencing strange sensations; feeling like “a third person is with us or in the car with us.” BP still spiking but more stable (highest 170 systolic)

Visit 9 (10 weeks)	0.5 g	80 mg (20 mg QID)	24 mg	900 mg (300 mg TID)	0.3 mg (0.1 mg TID)	30 mg (10 mg TID)	Tried to decrease to 0.25 g of phenibut but experienced dizziness, anxiety, and “little jolts” in his body while in bed. Also reports difficulty with memory. BP spiked up to 190 s systolic at times

Visit 10 (12 weeks)	0.25 mg	80 mg (20 mg QID)	24 mg	900 mg (300 mg TID)	0.3 mg (0.1 mg TID)	—	Feeling well. Not taking propranolol anymore

Visit 11 (14 weeks)	0.25 mg	80 mg (20 mg QID)	24 mg	900 mg (300 mg TID)	0.3 mg (0.1 mg TID)	—	Reports “I am doing better slowly.” BP elevated but stable. No longer spiking as high. Dizziness improving

Visit 12 (17 weeks)	One-third of scoop	80 mg (20 mg QID)	24 mg	900 mg (300 mg TID)	0.3 mg (0.1 mg TID)	—	Tried stopping phenibut but started sweating and experiencing neck tightness. BP spiking every afternoon

Visit 13 (19 weeks)	“Spritz of powder”	80 mg (20 mg QID)	24 mg	900 mg (300 mg TID)	0.3 mg (0.1 mg TID)	—	Basically off phenibut. “Doing okay.” BP still spiking, but reports not taking medications as scheduled

Visit 14 (21 weeks)	—	80 mg (20 mg QID)	22 mg	900 mg (300 mg TID)	0.3 mg (0.1 mg TID)	—	Off phenibut for 3 days now. Also started to taper lorazepam. “Doing fine.” BP stable

## Data Availability

The data that support the findings of this study are available within the manuscript under Table 1.
